# Characterization of the Host Factors Required for Hepadnavirus Covalently Closed Circular (ccc) DNA Formation

**DOI:** 10.1371/journal.pone.0043270

**Published:** 2012-08-13

**Authors:** Haitao Guo, Chunxiao Xu, Tianlun Zhou, Timothy M. Block, Ju-Tao Guo

**Affiliations:** 1 Department of Microbiology and Immunology, Drexel University College of Medicine, Doylestown, Pennsylvania, United States of America; 2 Institute for Hepatitis and Virus Research, Hepatitis B Foundation, Doylestown, Pennsylvania, United States of America; Yonsei University, Republic of Korea

## Abstract

Synthesis of the covalently closed circular (ccc) DNA is a critical, but not well-understood step in the life cycle of hepadnaviruses. Our previous studies favor a model that removal of genome-linked viral DNA polymerase occurs in the cytoplasm and the resulting deproteinized relaxed circular DNA (DP-rcDNA) is subsequently transported into the nucleus and converted into cccDNA. In support of this model, our current study showed that deproteinization of viral double-stranded linear (dsl) DNA also took place in the cytoplasm. Furthermore, we demonstrated that Ku80, a component of non-homologous end joining DNA repair pathway, was essential for synthesis of cccDNA from dslDNA, but not rcDNA. In an attempt to identify additional host factors regulating cccDNA biosynthesis, we found that the DP-rcDNA was produced in all tested cell lines that supported DHBV DNA replication, but cccDNA was only synthesized in the cell lines that accumulated high levels of DP-rcDNA, except for NCI-H322M and MDBK cells, which failed to synthesize cccDNA despite of the existence of nuclear DP-rcDNA. The results thus imply that while removal of the genome-linked viral DNA polymerase is most likely catalyzed by viral or ubiquitous host function(s), nuclear factors required for the conversion of DP-rcDNA into cccDNA and/or its maintenance are deficient in the above two cell lines, which could be useful tools for identification of the elusive host factors essential for cccDNA biosynthesis or maintenance.

## Introduction

Hepadnaviruses replicate their genomic DNA *via* protein-primed reverse transcription of RNA intermediates called pregenomic (pg) RNA in the cytoplasmic nucleocapsids [1]. The genomes of hepadnaviruses are relaxed circular (rc) partially double stranded DNA with viral DNA polymerase protein covalently attached to the 5′ terminus of minus strand DNA [2], [3], [4]. Upon entry into hepatocytes, the nucleocapsid delivers the genomic rcDNA into the nucleus, where the rcDNA is converted into covalently closed circular (ccc) DNA. cccDNA exists as an episomal minichromosome, and serves as the template for the transcription of viral RNAs [5].

Hepadnavirus DNA replication begins with viral DNA polymerase (pol) binding to a stem-loop structure (ε) near the 5′ end of pregenomic (pg) RNA, which primes viral minus stranded DNA synthesis and triggers the assembly of pgRNA/pol complex into nucleocapsid particle, where the pgRNA is reverse transcribed to produce minus strand DNA [6], [7]. The plus strand DNA is subsequently synthesized with a RNA primer derived from the terminal 18 ribonucleotides of the 5′ end of the pgRNA, which is translocated from the 3′ end of minus strand DNA to duplex with the DR2 sequence near the 5′ end of minus strand DNA to initiate plus-strand synthesis [8]. The subsequent template switch circularizes viral DNA to yield a faithful copy of the infecting viral rcDNA [9]. Occasionally, failure of primer translocation results in *in situ* priming of plus strand DNA synthesis at the 3′ end of minus strand DNA to produce dslDNA, which occurs during replication of wildtype hepadnaviruses at a frequency of about 5% [10].

In addition to incoming virion DNA, cccDNA can also be produced from newly synthesized cytoplasmic core DNA through an intracellular amplification pathway during the early phase of infection [11], [12]. These two pathways culminate in the formation of a regulated steady-state population of 5 to 50 cccDNA molecules per infected hepatocyte [5], [13], [14]. The longevity of cccDNA is still in debate. However, therapeutic elimination of cccDNA with highly active viral DNA polymerase inhibitors has not been achieved in chronically HBV-infected patients, and remains a major challenge for a cure to chronic hepatitis B [15], [16], [17], [18]. Better understanding of the molecular mechanism of cccDNA biosynthesis and maintenance should facilitate the development of novel therapeutic means to control chronic HBV infections [19].

Synthesis of cccDNA from rcDNA present in the incoming or newly synthesized core particles in the cytoplasm requires transport of rcDNA into the nucleus, capsid disassembly and conversion of rcDNA into cccDNA. However, where and how these molecular events take place remains largely elusive [20], [21]. Considering the structural feature of core-associated rcDNA, removal of viral DNA polymerase from the 5′ terminus of minus strand DNA ought to be an essential step in cccDNA biosynthesis. Indeed, we and others demonstrated previously that the hypothetic deproteinized rcDNA (DP-rcDNA) species existed in the virally infected hepatocytes *in vivo* and transfected hepatoma cells in cultures [21], [22]. Detailed characterization of DP-rcDNA had led us to propose a working model of cccDNA biosynthesis pathway [21], [23]. Briefly, further synthesis of plus strand DNA toward completion triggers the removal of genome-bound polymerase protein and nucleocapsid structure change, which leads to the exposure of a nuclear localization signal (NLS) at the carboxyl-terminus of capsid protein. The NLS in turn mediates the importation of the DP-rcDNA containing capsid into the nucleus [24], [25], [26]. Subsequently, the DP-rcDNA is converted into cccDNA by cellular DNA repair machinery [20].

One of the technical caveats in the previous study of DP-rcDNA is that the isolated nuclear DP-rcDNA was always contaminated with variable amounts of nicked cccDNA that was generated during preparation, which interfered with quantitative analysis of rcDNA deproteinization [21], [22]. To gain a better understanding of the molecular pathway of cccDNA synthesis, we intended to specifically investigate the deproteinization of hepadnaviral double-stranded linear (dsl) DNA species, which should avoid the interference of nicked cccDNA. To this end, we established a HepG2-derived stable cell line supporting replication of duck hepatitis B virus (DHBV) carrying G2552C mutation in a tetracycline inducible manner. The mutant virus predominantly synthesizes plus strand DNA *via in situ* priming, which results in the production of dslDNA, instead of rcDNA [10], [27]. It was demonstrated previously that unlike rcDNA, which formed cccDNA through faithful repair of the nicks in both plus and minus strand DNA, the dslDNA was converted into either cccDNA with deletions or insertions around the junction site, or oligomeric forms in which monomers were joined near the ends in random orientation, apparently *via* intra- or inter-molecular recombination [27], [28]. It is also known that the dslDNA is the predominant precursor of integrated viral DNA [29], [30]. Interestingly, similar with deproteinization of rcDNA, we observed in the current study that deproteinized dslDNA (DP-dslDNA) appeared 24 h earlier in the cytoplasm than cccDNA in the nucleus, suggesting that deproteinization of dslDNA also primarily takes place in the cytoplasm. Moreover, we demonstrated that Ku80, a sensory component of the non-homologous end joining (NHEJ) DNA repair pathway [31], is essential for the synthesis of cccDNA from dslDNA, but not from rcDNA. In addition, by testing a panel of 24 cell lines derived from different species and cell types for their ability to support DHBV cccDNA formation, we obtained evidence suggesting that deproteinization of rcDNA is most likely catalyzed by a viral or ubiquitous host function, but synthesis and/or maintenance of cccDNA requires specific nuclear factor(s), which is deficient in certain cell types.

Our findings presented herein thus shed light on the key steps of cccDNA biosynthesis, and provide basis for the identification of host factors essential for cccDNA formation, which should ultimately contribute to the development of novel intervention strategies to control chronic HBV infection.

## Results

### Establishment of Cell Lines producing hepadnaviral dslDNA

It has been shown previously that cccDNA can be produced from both rcDNA and dslDNA in hepadnavirus-infected cells [11], [27]. However, while rcDNA is faithfully repaired to form wild-type cccDNA, the cccDNA derived from dslDNA carries deletions and/or insertions around the site of end joining [27]. These observations suggest that rc- and dsl-DNA are converted into cccDNA *via* distinct mechanisms. In order to dissect the molecular pathway governing the synthesis of cccDNA from dslDNA, we took advantage of a previous observation that the mutation (G2552C) lying 6 nucleotides downstream of DR1 impeded the primer translocation step and resulted in predominant production of dslDNA [10], a HepG2-derived stable cell line supporting tetracycline-inducible replication of DHBV genome carrying G2552C mutation was established and designated as DSL212.

**Figure 1:**
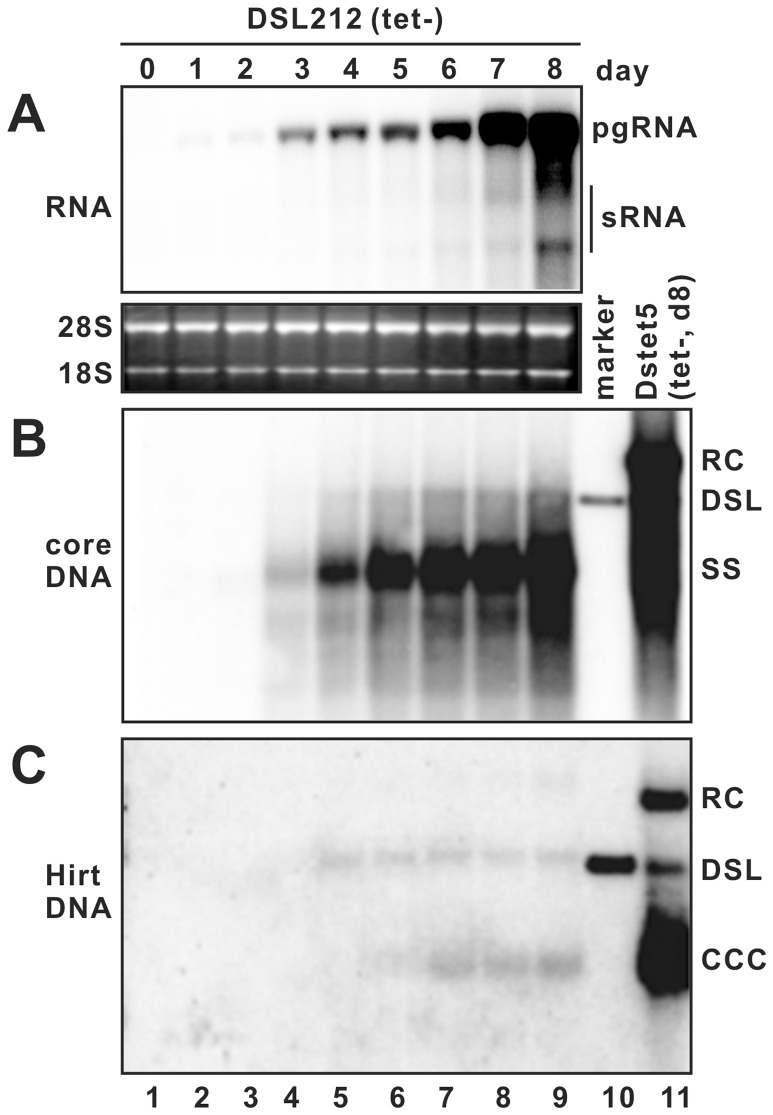
Kinetics of DHBV RNA transcription, DNA replication and cccDNA formation in DSL212 cells. DSL212 cells were seeded in 6-well plates and cultured in the presence of tetracycline (1 μg/ml) until cell monolayers became confluent. Cells were then cultured in media without tetracycline and harvested at the indicated days since the removal of tetracycline. Total cellular RNA, cytoplasmic core DNA and total cellular Hirt DNA were extracted and analyzed by Northern and Southern blot hybridization, respectively. (A) For viral RNA analysis, each lane was loaded with 5 μg of total RNA. pgRNA, pregenomic RNA; sRNA, mRNAs specifying the two envelope proteins. Ribosomal RNA (28S and 18S) served as loading controls. For DHBV core DNA (B) and Hirt DNA (C) analysis, each lane represents the amount of viral DNA extracted from one half of cells in a well of 6-well plate. RC, relaxed circular DNA; DSL, double stranded linear DNA; SS. single stranded DNA; cccDNA, covalently-closed circular DNA. Unit length of linear DHBV DNA (lane 10) and core or Hirt DNA extracted from dstet5 cells [40] cultured in the absence of tetracycline for 8 days (lane 11) served as controls.

As shown in Fig. 1A, DHBV pgRNA became detectable at one day after removal of tetracycline and continued increasing through day 1 to day 8. Full-length minus stranded DHBV DNA, dslDNA and cccDNA became readily detectable at day 3, day 4 and day 5, respectively. As predicted, rcDNA was not produced in DSL212 line (Fig. 1B). For cccDNA extraction from DSL212 cells, we made use of Hirt extraction which only isolates cellular extrachromosoal DNA without covalently bound proteins [32]. As shown in Fig. 1C, in addition to cccDNA, there is a DNA species that migrates at the same position with unit-length linear DNA in Hirt preparation, which should be free of covalently genome-bound viral DNA polymerase and thus the deproteinization product of core dslDNA (designated as DP-dslDNA). Interestingly, DP-dslDNA appeared at the same time as did the mature core-associated dslDNA at day 4 after tetracycline removal, suggesting that deproteinization of dslDNA occurred promptly upon its maturation.

### Deproteinization of dslDNA takes place in the cytoplasm

**Figure 2:**
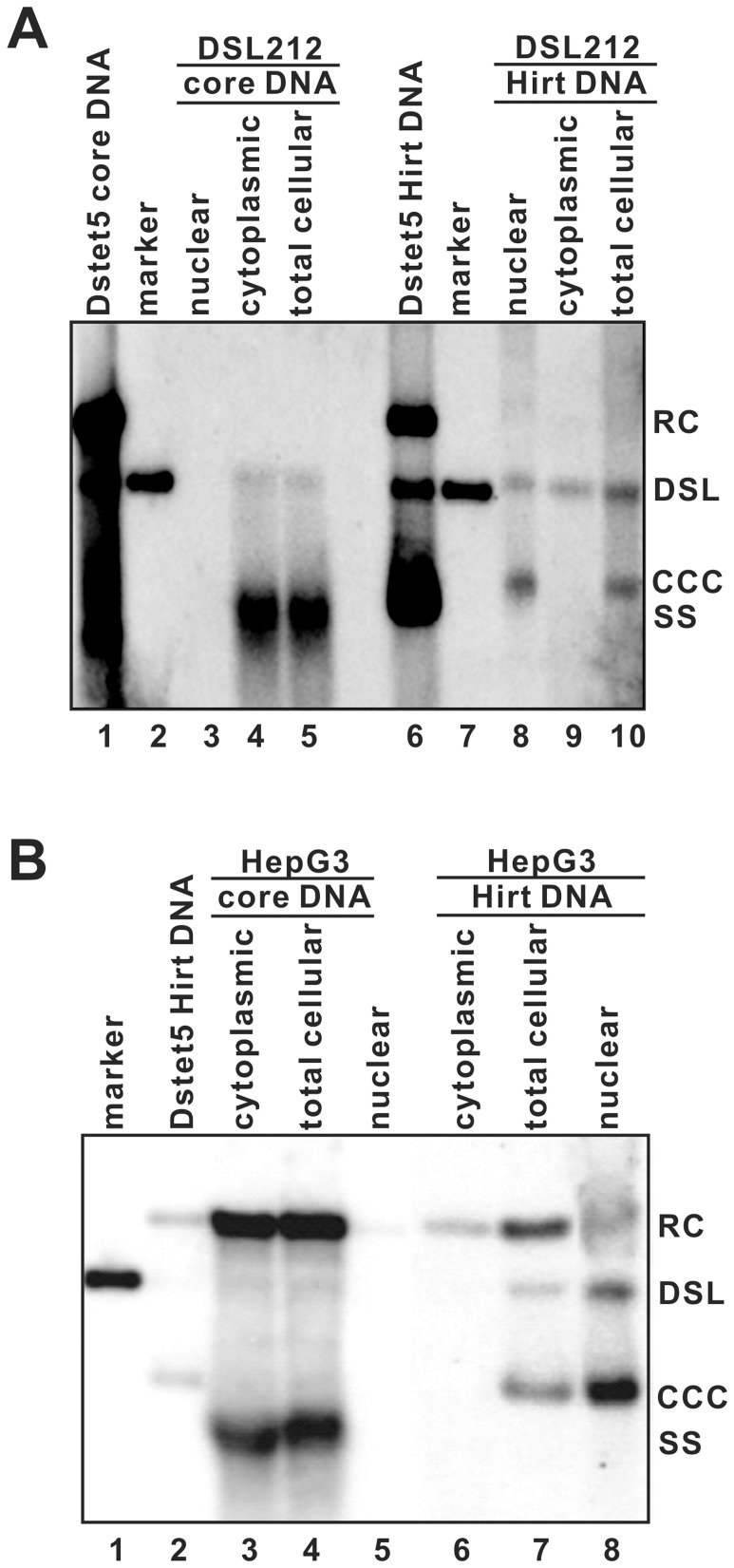
Subcellular distribution of DHBV DNA replication intermediates in HepG2 cells. (A) DSL212 cells were cultured in the absence of tetracycline for 6 days. Cytoplasm and nuclei were fractionated with QIAgen Qproteome Cell Compartment Kit by following the manufacturer's directions. DHBV core-associated DNA and Hirt DNA were extracted from whole cell, cytoplasm and nuclear fractions were analyzed by Southern blot assay. (B) DHBV core-associated DNA and Hirt DNA were extracted from whole cell, cytoplasm and nuclear fractions of HepG3 cells (a HepG2-derived stable cell line containing an integrated DHBV head-to-tail unit-length DNA dimer) were analyzed by Southern blot assay.

In order to investigate whether the deproteinization reaction of dslDNA occurs in the cytoplasm or nucleus, cell fractionation studies were performed. DSL212 cells were cultured in the absence of tetracycline for 10 days. HepG3, a HepG2-derived stable cell line containing an integrated wild-type DHBV head-to-tail unit-length genomic DNA dimer (unpublished data), was used as a control. Cytoplasmic and nuclear lysates were prepared from DSL212 and HepG3 cells with QIAgen Qproteome Cell Compartment Kit. Western blot analysis of cytoplasmic protein (annexin I) and nuclear marker (lamin A/C) in the cell fractions confirmed that there was no cross contamination between the cytoplasmic and nuclear fractions (data not shown). Intracellular capsid DNA and Hirt DNA extracted from whole cell, cytoplasmic and nuclear lysates were analyzed by Southern blot hybridization. As expected, cccDNA was detected only in the nuclear fraction of DSL212 and HepG3 cells (Fig. 2). Consistent with previous observations [21], [23], DP-rcDNA existed in both the cytoplasmic and nuclear fractions of HepG3 cells (Fig. 2B). Similar with DP-rcDNA, DP-dslDNA was found in similar amounts in the cytoplasmic and nuclear fractions of DSL212 cells (Fig. 2A).

Taken together, the results presented herein suggest that like rcDNA, removal of genome-bound viral DNA polymerase from dslDNA most likely occurs in the cytoplasm and the resulting DP-dslDNA is subsequently transported into the nucleus to convert into cccDNA.

### NHEJ DNA Repair Pathway is Essential for cccDNA Biosynthesis from dsl, but not rc DNA

**Figure 3:**
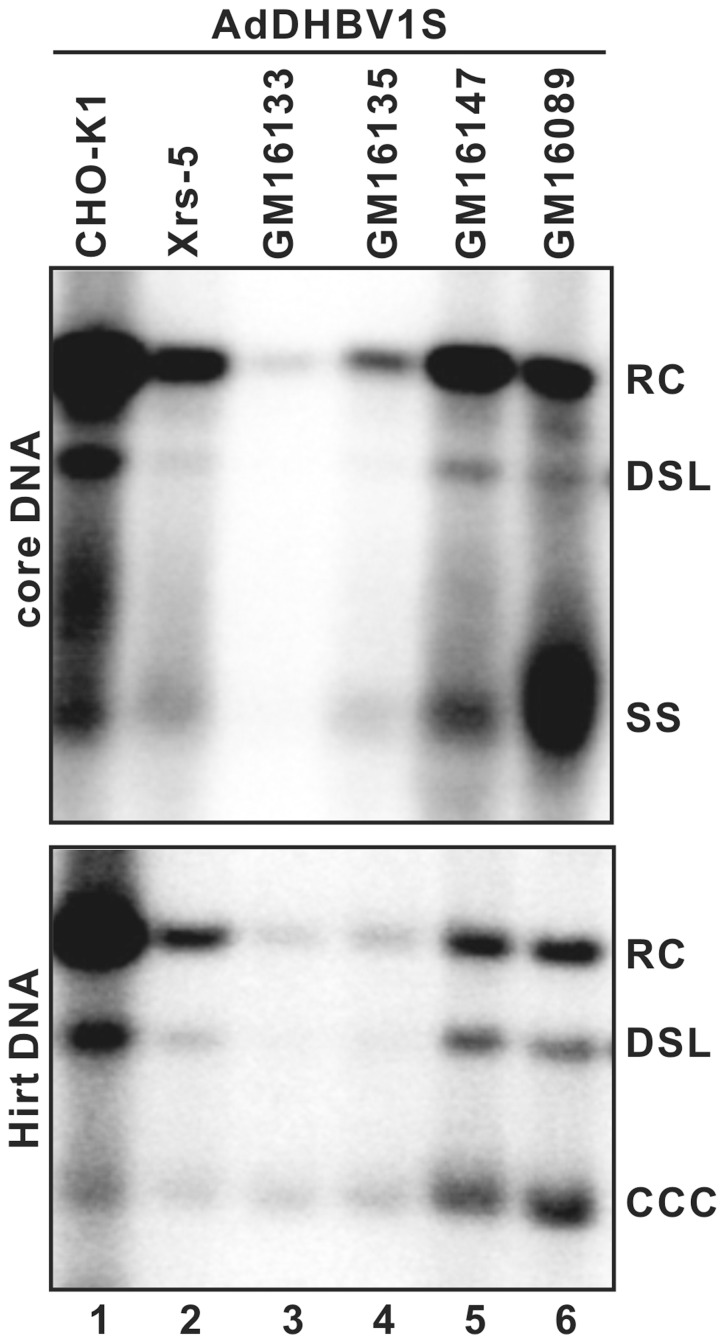
DHBV DNA replication and cccDNA formation in a panel of cells lines that are defective in NHEJ DNA repair pathway. Cell lines used in this experiment are CHO-K1 and its derived cell line Xrs-5 harboring defective gene of the p86 subunit of the Ku autoantigen, four human fibroblast cell lines GM16133, GM16135, GM16147 and GM16089 that are defective in XRCC1, the catalytic subunit of DNA-PK, XRCC4 and ligase IV, respectively. The cells are infected with AdDHBV1S at a MOI of 10 and infected cells are harvested on day 3 post infection. DHBV core-associated (upper panel) and Hirt DNA (lower panel) were extracted and analyzed by Southern blot hybridization.

Previous studies showed that while the nicks in both plus and minus strands of rcDNA were perfectly repaired to yield wild-type cccDNA, the cccDNA formed from dslDNA carried deletion or insertion around the site of end joining [27], [28]. These observations suggest that conversion of dslDNA, but not rcDNA, into cccDNA is through intra-molecular non-homologous recombination, which is most likely processed by the host cellular non-homologous end joining (NHEJ) DNA repair machinery. To test this hypothesis, four human skin fibroblast and one CHO cell lines deficient in specific genes required for NHEJ DNA repair were employed in this study, specifically GM16133 (XRCC1-deficient), GM16135 (DNA-PKcs-deficient), GM16147 (XRCC4-deficient), GM16089 (ligase IV-deficient), and Xrs-5 (Ku80-deficient). The above cell lines were infected with a recombinant adenoviral vector expressing an envelope-null (1S mutant) DHBV pgRNA under the control of CMV-IE promoter (AdDHBV1S) [33]. As shown in Fig. 3, both wild-type CHO (CHO-K1) cells and NHEJ-deficient cell lines supported DHBV DNA replication and cccDNA formation, albeit at a variable efficiency. Because rcDNA, but not dslDNA, is the predominant mature viral DNA form in these AdDHBV1S infected cell lines, the results are consistent with the notion that conversion of rcDNA into cccDNA is independent of the NHEJ DNA repair pathway.

**Figure 4:**
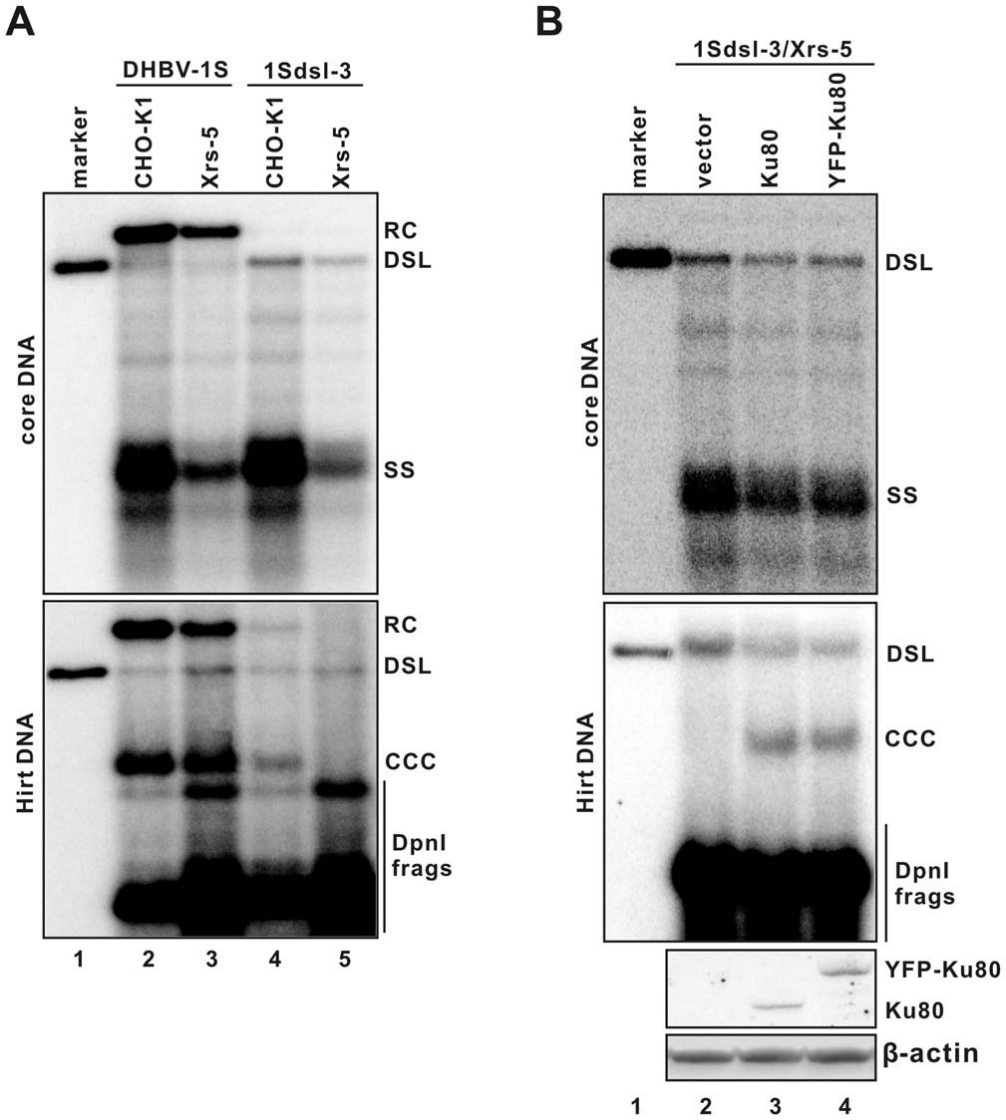
Role of Ku80 in DHBV cccDNA formation from dslDNA precursors. (A) CHO-K1 and Xrs-5 cells were transfected with plasmid DHBV-1S or 1Sdsl-3, respectively. On the day five post transfection, the cells were harvested and DHBV core-associated (upper panel) and Hirt DNA (lower panel) were extracted and analyzed by Southern blot hybridization. Unit-length DHBV genomic DNA served as a molecular weight control. (B) Xrs-5 cells were co-transfected with plasmid 1Sdsl-3 and vector plasmid pUC119 (lane 2), plasmid expressing wild-type Ku80 or Ku80-YFP fusion protein, respectively. The cells were harvested on day 5 post transfection. Core (upper panel) and Hirt (middle panel) DNA were detected by Southern blot hybridization. Ku80 and Ku80-YFP expression were detected by Western blot assay (lower panel). β-actin served as a loading control.

To specifically investigate the role of NHEJ pathway in cccDNA synthesis from dslDNA, wild-type CHO-K1 and a CHO-derived cell line deficient in Ku80 gene (Xrs-5) were transfected with a plasmid specifying either envelope protein-deficient (DHBV-1S) or 1S/G2552C mutant (1Sdsl-3) DHBV pregenome. Three days after transfection, the cells were harvested and DHBV core-associated DNA and Hirt DNA were extracted and analyzed by Southern blot hybridization. As shown in Fig. 4A, both cell lines supported efficient replication of DHBV 1S and 1S/G2552C mutant genomes. As expected, only dslDNA, but not rcDNA, was detected in 1Sdsl-3 transfected cells. However, while cccDNA could be detected in CHO-K1 cells transfected with either plasmid, it was only detectable in Xrs-5 cells transfected with plasmid DHBV-1S, but not 1Sdsl-3. The result thus suggested that Ku80 was essential for cccDNA biosynthesis from dsl, but not rc DNA. In an effort to further confirm this observation, we demonstrated that expression of either a wild-type or a functional Ku80-yellow fluorescent protein (YFP) fusion protein in Xrs-5 cells was able to restore the ability of the cell line to support cccDNA formation from dslDNA (Fig. 4B). Hence, the results presented herein firmly established an essential role of Ku80, and the NHEJ DNA repair pathway by inference, in cccDNA synthesis from dslDNA, but not rcDNA.

Intriguingly, we noticed that failure to form cccDNA in the plasmid 1Sdsl-3-transfected Xrs-5 cells resulted in accumulation of DP-dslDNA, and restoration of cccDNA synthesis by Ku80 expression was accompanied with a reduction of DP-dslDNA level (Fig. 4B, comparing lane 2 with lanes 3 and 4 in Hirt DNA gel). This observation thus reinforces our previous hypothesis that deproteinized rc and dsl DNA are direct precursors of cccDNA biosynthesis [21], [23].

### Cell Line Specific Nuclear Function Is Required for cccDNA Formation

**Figure 5:**
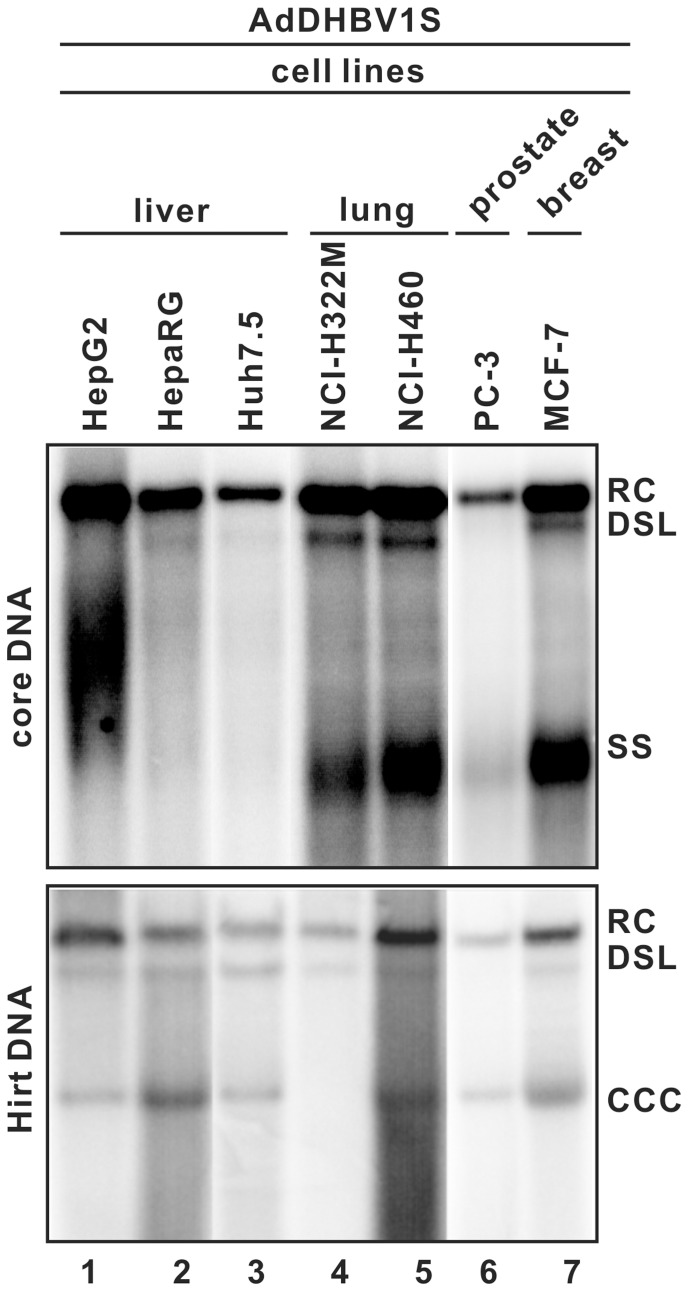
DHBV DNA replication and cccDNA formation in a panel of human cell lines infected by AdDHBV1S. The indicated cell lines were seeded onto 6-well plate and infected with AdDHBV1S at a MOI of 10. Five days post infection, DHBV core-associated (A) and Hirt DNA (B) were extracted and analyzed by Southern blot hybridization. Each lane represents the amount of viral DNA extracted from one half of cells in a well of 6-well plate.

**Figure 6:**
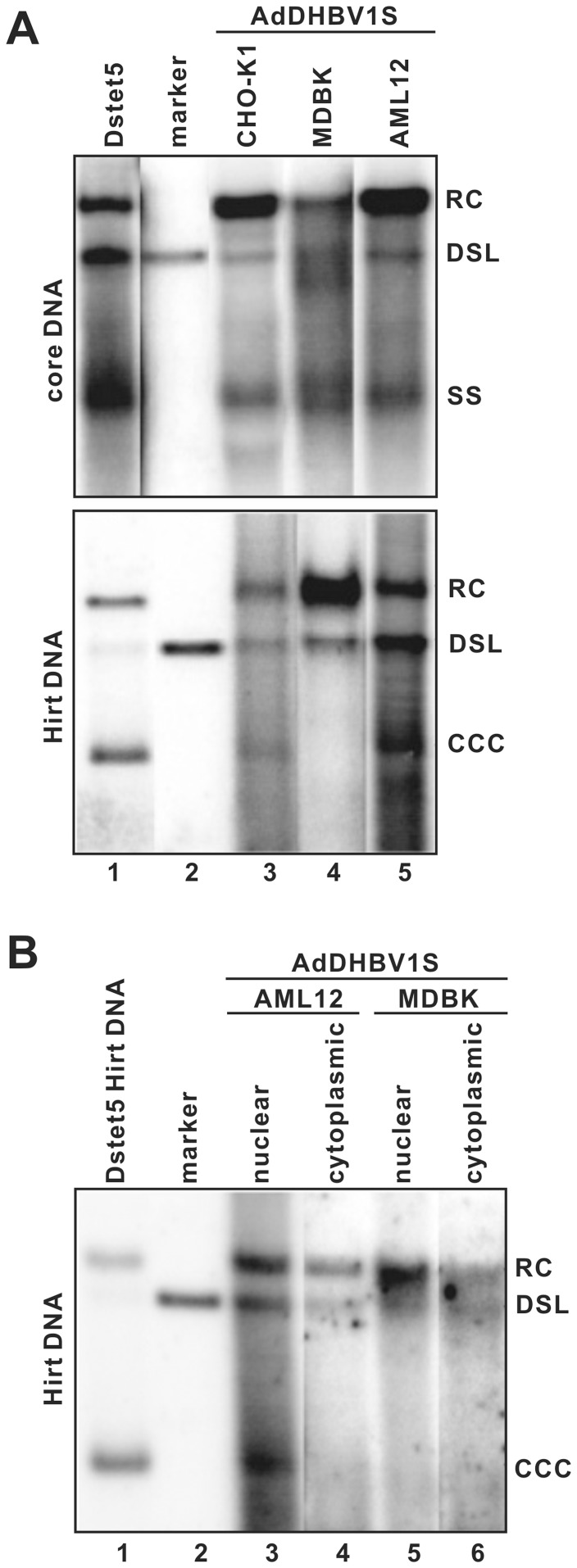
Subcellular distribution of the DP-DNA in MDBK and NCI-H322M cells. (**A**) AML12 and MDBK were infected with AdDHBV1S at a MOI of 10. On day 5 post infection, DHBV core-associated (upper panel) and Hirt DNA (lower panel) were extracted and analyzed by Southern blot hybridization. Hirt DNA prepared from the cytoplasmic and nuclear fractions of AdDHBV1S-infecetd AML12 and MDBK cells (B) or NCI-H322M cells (C) were analyzed by Southern blot hybridization assay. Core or Hirt DNA extracted from dstet5 cells cultured in the absence of tetracycline for 8 days (lane 1) and unit length of linear DHBV DNA (lane 2) served as controls.

Previous studies suggested that hepadnavirus cccDNA formation in hepatocyte-derived cell lines was regulated in a virus-specific manner [22], [34]. In comparison with HBV, DHBV cccDNA was more efficiently produced in both human and avian hepatoma cells. The low cccDNA productivity of HBV is generally attributed to its inefficiency in the conversion of DP-rcDNA into cccDNA in the nucleus [34]. In addition, cccDNA formation was also observed in HEK293 cells transfected with plasmids expressing HBV or DHBV pgRNA under the control of a CMV IE promoter [22], together with data presented above (Figs. 3 and 4), suggesting that cccDNA biosynthesis could take place in non-hepatocyte-derived cells. In a search for host cellular factors regulating cccDNA formation, we tested a panel of 24 cell lines derived from different species and cell types for their ability to support cccDNA formation upon infection with AdDHBV1S. As summarized in Table 1 and by the representative results shown in Figs. 5 and 6A, DHBV core-associated DNA replication intermediates can be detected in all the cell lines tested, except for LNCAP (a human prostate cancer cell line) and L929 (a mouse fibroblast cell line) due to the loss of cell viability caused by adenoviral infections. Interestingly, the results from the Hirt DNA analysis clearly demonstrated that DP-rcDNA was produced in all the 22 cell lines that supported DHBV DNA replication. With a few exceptions, the amount of DP-rcDNA in a given cell line was correlated with the amount of core DNA. In conjunction with our previous observation that rcDNA deproteinization could occur within either virion-derived or purified intracellular nucleocapsids following an endogenous DNA polymerase reaction [23], our results seem to suggest that the removal of genome-bound viral DNA polymerase is catalyzed by either a viral or ubiquitous host factor encapsidated in nucleocapsid.

**Table 1:** Cell Permissiveness on DHBV replication, deproteinization and cccDNA formation.

AdDHBV1S infected cell lines	rcDNA	DP-rcDNA	cccDNA
***Human liver cancer cell***			
HepG2	**++++** ***^a^***	**++++** *^a^*	**++++** *^a^*
HepaRG	**+++**	**+++**	**++++++++**
Huh7	**+**	**+**	**++**
Huh7.5	**++**	**++**	**++++**
Hep3B	**+**	**+**	-*^b^*
***Human lung cancer cell***			
NCI-H322M	**++++**	**+++**	-
NCI-H460	**++++**	**++++**	**++++++++**
NCI-H520	**+++**	**+**	-
A549	**++++**	**++++**	**++++**
NCI-H23	**+**	**+**	-
NCI-H226	**+**	**+**	-
***Human colon cancer cell***			
HCT-15	**+**	**+**	**-**
SW620	**+**	**+**	**-**
***Human prostate cancer cell***			
PC3	**++**	**++**	**++++**
LNCaP	ND*^c^*	ND	ND
***Human breast cancer cell***			
MCF-7	**++++**	**++++**	**+++++++**
MDA-MB-231	**+**	**+**	**-**
***Human Ovarian cancer cell***			
OVCAR-3	**+**	**+**	**-**
***Human cervical cancer cell***			
Hela	**+**	**+**	**-**
***Immortalized non-human cell***			
AML12 (mouse hepatocyte)	**+++++**	**++++**	**++++++**
L929 (mouse fibroblast cell)	ND	ND	ND
Vero (monkey kidney cell)	**+++++**	**++++++++**	**++++++++++**
MDBK (bovine kidney cell)	**++**	**+++++++**	**-**
CHO-K1 (hamster ovarian cell)	**+++++**	**++**	**+++**

Note: *^a^* each “**+**” represents one quarter of quantitative signal for each DHBV DNA species from HepG2 cells by DNA hybridization; *^b^* undetectable by DNA hybridization; *^c^* not determined due to cell death after AdDHBV1S infection.

However, in contrast with the efficient and ubiquitous production of DP-rcDNA, cccDNA could only be detected in 11 out of 13 cell lines that supported high level DNA replication. Interestingly, although cccDNA was not synthesized in mouse hepatocytes that supported efficient HBV DNA replication and accumulation of DP-rcDNA [35], [36], DHBV cccDNA was readily detected in AdDHBV1S transduced AML-12 cells (immortalized mouse hepatocytes) (Fig. 6A). These observations further support the notion that hepadnavirus cccDNA biosynthesis is regulated in a viral specific fashion [34]. Moreover, it appeared that not only human and mouse hepatoma cells, but also selected human lung, prostate and breast cancer cell lines, fibroblasts and epithelia (Vero and CHO-K1) derived from other species supported efficient cccDNA formation. These observations imply that host factors required for hepadnavirus cccDNA synthesis are not hepatocyte-specific, but expressed in a wide variety of cell types. Surprisingly, despite high levels of core DNA and DP-rcDNA were accumulated in NCI-H322M and MDBK cells, cccDNA can not be detected in these two cell lines (table 1, Figs. 5 and 6A). To map the step(s) limiting the cccDNA formation, we determined the subcellular distribution of DP-rcDNA in these two cell lines. Our results showed that DP-rcDNA were readily detectable in both the cytoplasm and nuclei of MDBK (Fig. 6B) and NCI-H322M (data not shown), suggesting that not the DP-rcDNA nuclear importation pathway, but certain nuclear component(s) required for the conversion of DP-rcDNA into cccDNA are deficient in these two cell lines.

## Discussion

Synthesis of cccDNA is a critical, but not well-understood step in the life cycle of hepadnaviruses. Our current study further characterized the molecular pathway of cccDNA formation from dslDNA precursor and examined the role of host cellular NHEJ DNA repair pathway in cccDNA synthesis. The findings presented in this report have several important implications in hepadnavirus biology and development of antivirals to cure chronic hepatitis B.

First, using a cell line supporting the production of DHBV dslDNA, we investigated the molecular pathway of cccDNA biosynthesis from the dslDNA precursor. The time course and cell fractionation studies presented in Figs. 1 and 2 clearly demonstrated that similar with DP-rcDNA, the DP-dslDNA existed in both the cytoplasm and nuclei and appeared 24 h earlier than cccDNA. These observations further supported our hypothesis that the removal of covalently attached viral polymerase from hepadnaviral mature genome DNA takes place in the cytoplasm and the resulting DP-rc and -dslDNA are subsequently imported into the nuclei, where they are converted into cccDNA [21], [23].

**Figure 7:**
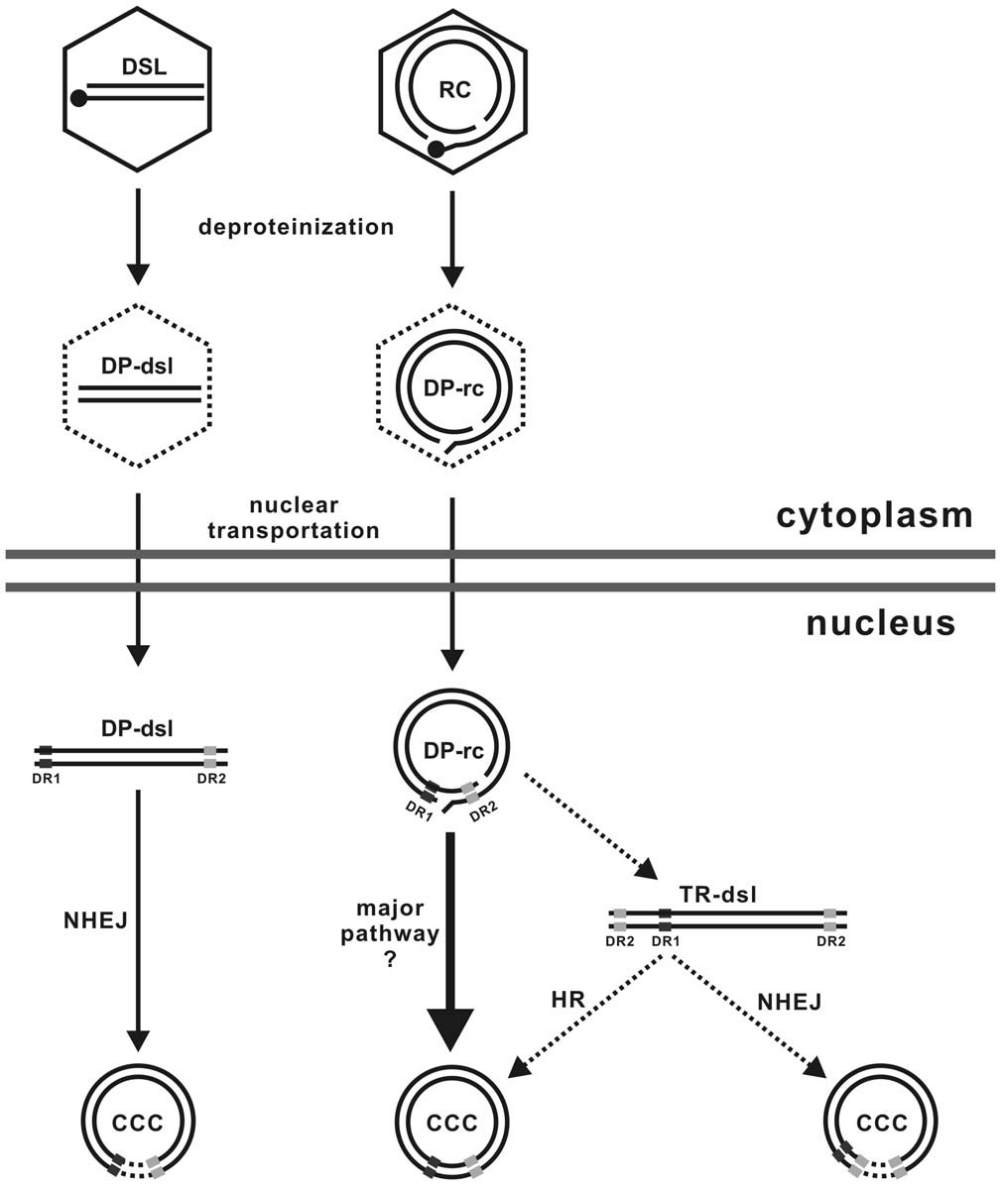
Schematic representation of cccDNA biosynthesis pathways from rc and dslDNA. See text for detailed explanation.

Second, as illustrated in Fig. 7, based on their unique structural features, DP-rcDNA and -dslDNA have been speculated to be converted into cccDNA by distinct cellular DNA repair machinery. While it is generally believed that multiple DNA repair components/pathways might participate in repair of the two gaps in rcDNA during cccDNA formation [20], it is also postulated that rcDNA may at first be converted into a double-stranded linear DNA containing terminal repeats (TR-dsl DNA) through extension of both plus and minus strand DNA over the cohesive-end region by viral and/or host DNA polymerases, and cccDNA is subsequently formed *via* intra-molecular homologous recombination of TR-dsl DNA [28]. Although the TR-dsl DNA is undetectable in virally infected hepatocytes by conventional hybridization methods, previous sequence analysis of cccDNA recombinant joints in the livers of DHBV-infected ducks and WHV-infected woodchucks provided evidence supporting that cccDNA could be formed from two types of linear DNA, the dslDNA derived from *in situ* priming and the putative TR-dsl DNA, through nonhomologous recombination [27], [28]. In this study, we vigorously confirmed that NHEJ pathway is indeed required for cccDNA formation from the dslDNA, but not rcDNA precursor.

Third, it appears that deproteinized rcDNA and dslDNA can be detected in all the cell types that successfully synthesize full-length DHBV rcDNA and dslDNA (Table 1, Figs. 5 and 6). Time course studies also indicated that DP-rc and -dslDNA always appeared simultaneously with the full-length core-associated rc and dslDNA [21] (Fig. 1). These observations imply that the key requirement for deproteinization to occur is the completion of plus strand DNA synthesis and the deproteinization reaction is most likely catalyzed by a viral or ubiquitous host factor encapsidated or associated with viral nucleocapsids. If this is indeed the case, proteomic analysis of purified core particles may be helpful to reveal the nature of host factor(s) involved in the removal of the polymerase from viral DNA. In addition, our study also suggested that although cccDNA can be efficiently formed in hepatocytes as well as many other cell lines derived from various cell types and species, certain host nuclear factors required for cccDNA synthesis were indeed deficient in a few cell lines, such as NCI-H322M and MDBK. Further characterization of these cell lines in comparison to the cccDNA permissive lines should facilitate the identification of the elusive host factors.

Fourth, detailed characterization of the distinct forms of hepadnaviral rcDNA and dslDNA accumulating in the cells, in the current and previous studies by us and others, strongly suggest that it is not the deproteinization of viral genomes and nuclear import of deproteinized rc and dslDNA, but the intranuclear conversion of deproteinized DNA into cccDNA that determines the efficiency of cccDNA synthesis [21], [22], [34]. However, it remains to be determined at this time whether this is due to the failure of the deproteinized viral DNA release from imported nucleocapsid (uncoating), or recognition and/or repair of deproteinized DNA by cellular DNA repair apparatus. Future investigation on nuclear viral DNA structure, nucleocapsid uncoating [34], [37], recognition, and response to nuclear viral DNA by cellular DNA repair machinery should provide answers to these important questions.

Finally, the fact that many cell types failed to support HBV and DHBV cccDNA formation strongly suggests that there are indeed host factors that are dispensable for cell viability, but absolutely required for cccDNA synthesis. Alternatively, it is also possible that the lack of cccDNA in some of these cells is not due to their inability to synthesize cccDNA, but deficiency of host factor(s) essential to maintain the episomal cccDNA in the nuclei. Nevertheless, identification of these host factors should advance our understanding of HBV biology and, more importantly, provide a basis for development of therapeutics to target such host factors, which should selectively suppress cccDNA synthesis and ultimately eliminate cccDNA from infected cells.

## Materials and Methods

### Cell Lines

HepG2, CHO-K1 and Xrs-5 cell lines were purchased from ATCC. HepaRG cells were purchased from Biopredic International (Rennes, France). Four human fibroblast cell lines GM16133, GM16135, GM16147 and GM16089 were purchased from CORIELL Institute for Medical Research (Camden, NJ). HepG3 is a HepG2-derived stable cell line containing an integrated DHBV head-to-tail unit-length genomic DNA dimmer and obtained from Dr. William S. Mason (Fox Chase Cancer Center, Philadelphia).

### Plasmids

Plasmid DHBV-1S directs the expression of envelope-null (1S) DHBV pgRNA under the control of the cytomegalovirus immediate early (CMV IE) promoter. The 1S mutant carries three termination codons in the envelope gene that prevent translation of both envelope proteins p17 and p36 [33]. A point mutation of G2552C was introduced into DHBV DNA in plasmid DHBV-1S with QuikChange II Site-Directed Mutagenesis Kit (Agilent Technologies, Santa Clara, CA) to yield plasmid 1Sdsl-3. The pregenomic RNA-coding sequence in plasmid 1Sdsl-3 was amplified by polymerase chain reaction and inserted into Not I- and Sal I-restricted plasmid pTRE2 (Clontech, Mountain View, CA) to yield plasmid pTREDHBV1Sdsl. The intended mutations in both plasmids were confirmed by sequence analysis. The plasmids expressing wild-type Ku80 or Ku80-YFP fusion protein were kindly provided by Dr. David J. Chen (University of Texas Southwestern Medical Center, Dallas, TX) [38].

### Construction of Recombinant Adenovirus

Replication deficient adenovirus AdDHBV1S was constructed using AdEasy-XL kit (Agilent Technologies). Briefly, a CMV-IE driven envelope-null (1S) DHBV pregenome-coding sequence derived from plasmid DHBV-1S was ligated into the multiple cloning site of pShuttle vector, digested with Pme I followed by transformation of BJ-5183-Ad-1 cell that harbors pAdEasy-1 vector. pAdEasy-1 is an ampicillin resistant 33.4 kb plasmid that contains all genes of adenovirus serotype 5 (Ad5) but E1 and E3. Recombinants of pAdEasy-DHBV1S were selected through kanamycin resistance and were linearized with Pac I. Five micrograms of linearized DNA were used to transfect AD-293 cell with Lipofectamine reagents (Life Technologies, Grand island, NY) in a T25 flask. Culture medium was replaced every 3 days, and viral plaques were observed 10–14 days post transfection. Cells were then collected with 1 ml culture medium and subjected to 3 freeze/thaw cycles in methanol/dry ice bath. Cellular debris was removed by centrifugation at 12,000g for 10 minutes and 5 μl of supernatant were used to inoculate AD-293 cells in T75 flask for further amplification of recombinant adenoviruses. The amplified AdDHBV1S were purified with Adeno-X Maxi purification Kit (Clontech).

To determine the ability of cells lines to support DHBV replication and cccDNA formation, cells were cultured in six-well plates and infected with AdDHBV1S at a multiplicity of infection (MOI) of 10. Three or five days post infection, DHBV core-associated and Hirt DNA were extracted and analyzed by Southern blot hybridization as described previously [39].

### Establishment of Stable Cell Line

HepG2 cells were transfected with plasmid pTet-off (Clontech) that expresses tet-responsive transcriptional activator (tTA) and plasmid pTREDHBV1Sdsl, in which DHBV pgRNA expression is controlled by a cytomegalovirus early promoter with tetracycline responsive element. Transfected HepG2 cells were selected with 500 μg/ml G418 in the presence of 1 μg/ml tetracycline. G418-resistant colonies were picked and expanded into cell lines. DHBV replication was induced by culturing cells in tetracycline-free medium, and the levels of viral DNA replicative intermediates were determined by Southern blot hybridization. The cell lines with high levels of DHBV replication were chosen and designated as DSL212.

### DHBV DNA and RNA Analyses

Intracellular viral core DNA was extracted as described previously [40], [41]. One half of the DNA sample from each well of 6-well plates was resolved by electrophoresis into a 1.5% agarose gel and transferred onto Hybond-XL membrane. Extraction of protein-free viral DNA was carried out by using a modified Hirt extraction procedure [32], [42]. Briefly, cells from one 35mm diameter dish were lysed in 3 ml of 10 mM Tris-HCl (pH 7.5), 10 mM EDTA and 0.7% SDS. After 5 minutes incubation at room temperature, the lysate was mixed with 1 ml of 2.5M KCl and incubated at room temperature for 30 min and followed by centrifugation at 10,000g for 15 min at 4°C. The supernatants were extracted twice with phenol, and once with phenol: chloroform. DNA was precipitated with two volumes of ethanol overnight at room temperature and dissolved in TE buffer (10 mM Tris-HCl, pH 8.0, 1 mM EDTA). One half of the protein-free DNA sample was then resolved in a 1.2% agarose gel and transferred onto Hybond-XL membrane. For viral RNA analysis, total cellular RNA was extracted with TRIzol reagents (Life Technologies). Five micrograms of total RNA was resolved in 1.5% agarose gel containing 2.2 M formadelhyde and transferred onto Hybond-XL membrane in 20X SSC buffer.

For the detection of DHBV DNA and RNA, membranes were probed with either a α-^32^P-UTP (800 Ci/mmol, Perkin Elmer) labeled minus or plus strand specific full-length DHBV riboprobe. Hybridization was carried out in 5 ml EKONO hybridization buffer (G-Biosciences, St. Louis, MO) with 1 hour pre-hybridization at 65^o^C and overnight hybridization at 65^o^C followed by a 1 hour wash with 0.1X SSC and 0.1% SDS at 68°C. The membrane was exposed to a phosphoimager screen and hybridization signals were scanned and quantified with QuantityOne software (Bio-Rad, Hercules, CA).

### Cell Fractionation

The cytoplasmic and nuclear fractions of DSL212, HepG3 and AdDHBV1S-infecetd MDBK or NCI-H322M cells were separated with Qproteome Cell Compartment Kit (QIAgen, Valencia, CA) by following the manufacturer's directions. Purity of the cytoplasmic and nuclear fractions was confirmed by measuring cytoplasmic and nuclear specific protein markers (Annexin I and Lamin A/C, respectively) with Western blot assay by following the manufacturer's procedures. Total and protein-free viral DNA were extracted from both nuclear and cytoplasmic fractions. Viral DNA were analyzed by Southern blot hybridization.

### Transient Transfection Assay

CHO-K1 and Xrs-5 cells were seeded into 35 mm diameter dishes at a density of 1.2×10^6^ cells per dish and cultured in antibiotics-free complete DMEM/F12 medium. One day post seeding, cells were transfected with plasmid DHBV-1S and 1Sdsl-3, respectively. Five days later, the cells were harvested and DHBV core-associated and Hirt DNA were extracted and analyzed by Southern blot hybridization.

### Western Blot Assay

Cells in one well of a 6-well-plate were washed once with PBS buffer and lysed in 300 μl of 1× Laemmli buffer. Thirty microliters of the cell lysate was resolved on a 10% SDS-PAGE and proteins were transferred onto Immobilon PVDF-FL membrane (Millipore, Billerica, MA). The membranes were blocked with Western Breeze blocking buffer (Life Technologies) and probed with antibodies against Ku80 (Kindly provided by Dr. David J. Chen)[38] or β-actin (Millipore). Bound antibodies were revealed by IRDye secondary antibodies and visualized using the Li-COR Odyssey system.
